# Structured Transition Protocol for Children with Cystinosis

**DOI:** 10.3389/fped.2017.00191

**Published:** 2017-08-31

**Authors:** Rupesh Raina, Joseph Wang, Vinod Krishnappa

**Affiliations:** ^1^Department of Pediatric Nephrology, Akron Children’s Hospital, Akron, OH, United States; ^2^Department of Nephrology, Cleveland Clinic Akron General, Akron, OH, United States; ^3^University of Nebraska Medical Center, Omaha, NE, United States; ^4^Lake Erie College of Osteopathic Medicine, Erie, PA, United States

**Keywords:** transition care, transition clinic, pediatric, adolescent, chronic disease self-management

## Abstract

The transition from pediatric to adult medical services has a greater impact on the care of adolescents or young adults with chronic diseases such as cystinosis. This transition period is a time of psychosocial development and new responsibilities placing these patients at increased risk of non-adherence. This can lead to serious adverse effects such as graft loss and progression of the disease. Our transition protocol will provide patients, families, physicians, and all those involved a structured guide to transitioning cystinosis patients. This structured protocol depends on four areas of competency: Recognition, Insight, Self-reliance, and Establishment of healthy habits (RISE). This protocol has not been tested and therefore challenges not realized. With a focus on medical, social, and educational/vocational aspects, we aim to improve transition for cystinosis patients in all aspects of their lives.

## Introduction

Pediatric patients with chronic diseases face a unique challenge when they reach adolescence and young adulthood (1). During this time, adolescents and young adults (AYA) are expected to transition from physician- and family-driven medical care to self-management of their disease (2). At the same time, AYA’s undergo psychological, social, and educational/vocational maturation (2). This maturation process leads to an increased risk of medication non-adherence, missed appointments, and other deficiencies detrimental to their medical health (2, 3). This can lead to serious adverse medical outcomes.

Nephropathic cystinosis is a rare autosomal recessive disorder resulting in life-long complications (4). It has an estimated incidence of 1 in 100,000–200,000 live births (5, 6). Pediatric patients inevitably develop kidney failure requiring transplant (7). Furthermore, an intensive life-long medication regimen is required to slow down disease progression in other organ systems (8). The combination of a chronic disease process, life-long complicated medication regimen, multi-organ involvement, and need for transplant places these patients at considerable risk during transition. As such, the *Cystinosis Research Network* has made transition research a priority (9). We have developed a transition protocol based on four key competency areas, Recognition, Insight, Self-reliance, and Establishment of healthy habits (RISE). Through the use of a structured transition protocol, we believe we can improve transition and cystinosis outcomes.

## Cystinosis

Defective or absent cystinosin leads to the accumulation of cystine within lysosomes resulting in the continuous development of cystine crystals (4, 7). This process occurs in all organ systems but typically affects the kidney first. Three forms of cystinosis exist: *Infantile, Juvenile*, and *benign-adult*. Infantile cystinosis is the most severe and common of the three accounting for 95% of all cases. Symptoms typically present within the first year of life and without treatment, renal failure will occur by the 10th year of life (5, 10). Juvenile form typically presents in early adolescence with renal failure by the third decade (5, 10). Adult onset is mildest of the three with no kidney involvement and onset is typically in middle age (5, 10).

**Figure 1:**
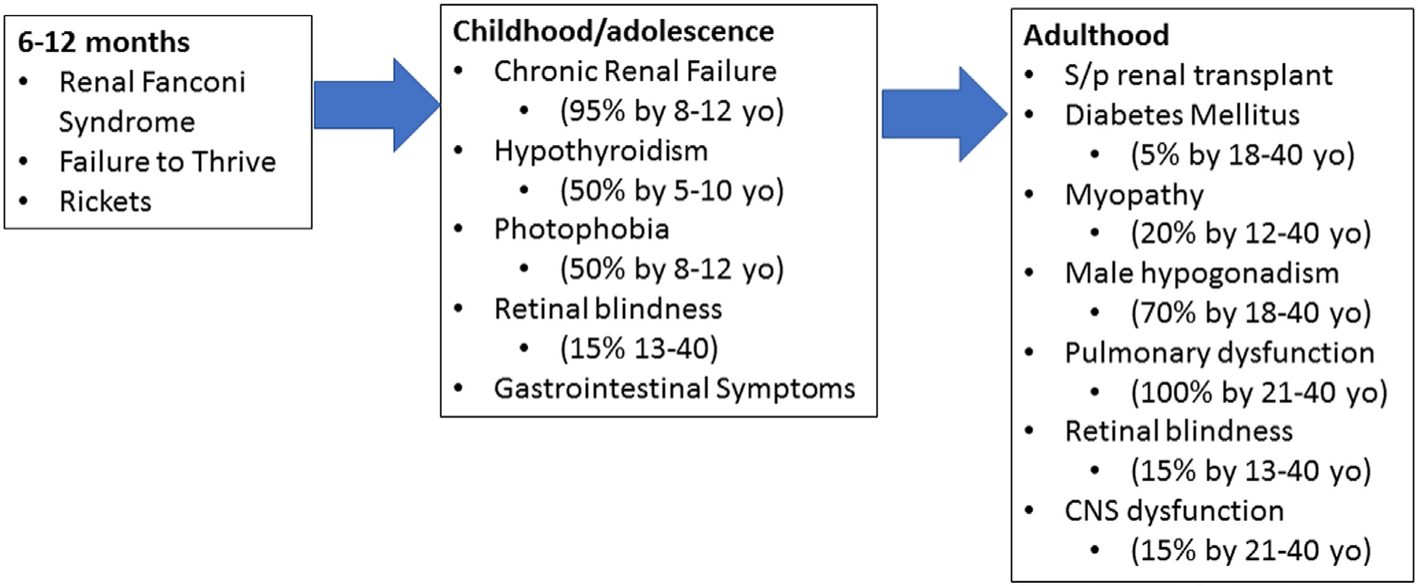
Progression of cystinosis. Cystinosis is a progressive disease with accumulation beginning during fetal life. As a result, signs and symptoms of cystinosis begin manifesting between 6 and 12 months of age. If left untreated, multiorgan dysfunction begins to appear in early childhood and adolescence. Even with adequate treatment, renal transplant is inevitable and typically occurs in adolescence or early adulthood. Without adequate treatment, cystinosis results in severe multiorgan damage even after renal transplantation.

Patients who suffer from infantile cystinosis begin accumulation during the fetal period (4). By 12 months of age, patients present with renal Fanconi syndrome, failure to thrive, rickets, and electrolyte imbalances (Figure 1) (4, 8). Failure to thrive, dehydration, and polyuria are the most common initial symptoms. Biochemically, they present with hypokalemia, hypophosphatemia, metabolic acidosis, and hypocalcemia (4, 8). By 1–2 years of age, corneal crystals can be appreciated on slit-lamp examination (4, 8). Diagnosis is confirmed by measuring cystine within white blood cells (WBCs), demonstration of corneal cystine crystals on slit lamp, or genetic analysis (7).

If allowed to continue without medical management, patients will proceed to develop chronic renal failure, photophobia due to cystine accumulation within the eye, and endocrine dysfunction by the first decade of life (4, 8). Patients will require a renal transplant at this stage and while curative from a kidney standpoint, cystine continues to accumulate in other organs. Retinal blindness, diabetes mellitus, muscle wasting, and other complications will occur during the first and second decade with death within this time frame as well (4, 8).

The advent of cystine-depleting treatment (cysteamine) has greatly improved morbidity and mortality in cystinosis. Early diagnosis, successful transplantation, and strict adherence to medications have now greatly prolonged the life span of cystinosis patients (8). With the increase in life span, more patients suffer from extrarenal complications as a result (11).

Kidney transplant is very effective and curative, in regards to kidney function, as the new kidney would not be affected by the patient’s disease (12). However, because cystine continues to accumulate in all other organs, it must be controlled in order to combat extrarenal manifestations of cystinosis (13). Therefore, despite successful transplantation, patients must continue their strict medication regimen. A typical medication regimen includes: cysteamine (oral), cysteamine eye drops, electrolyte and mineral replacement, organ rejection medications, endocrine hormone replacement, and many other medications (7–9). This complex regimen requires strict adherence in order to prevent further complications.

**Figure 2:**
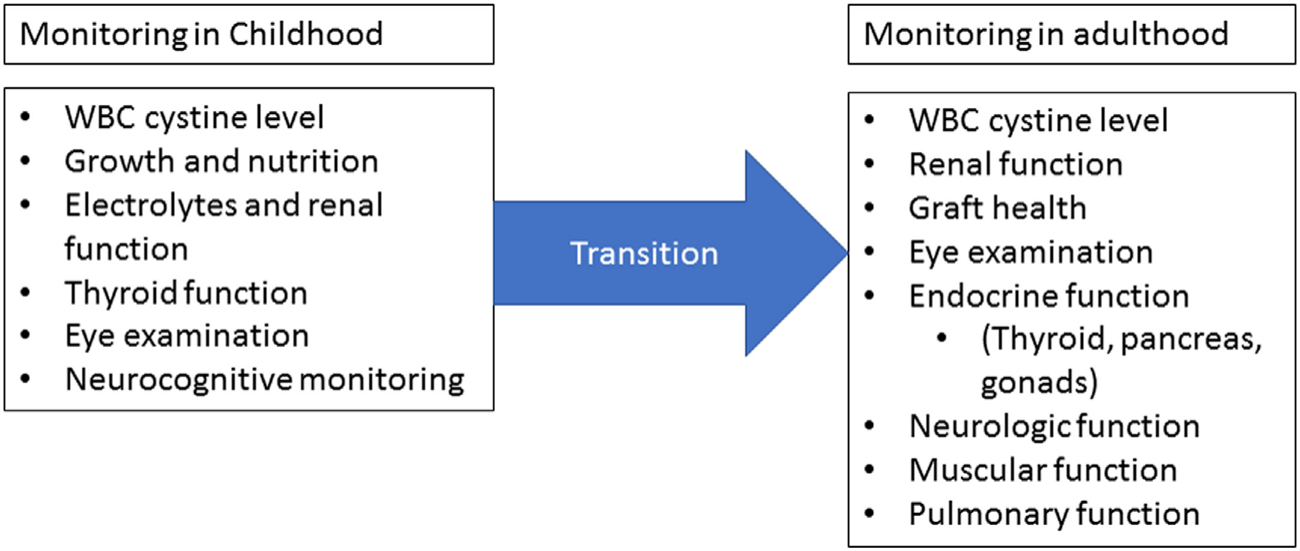
Monitoring requirements. Once cystinosis has been diagnosed, routine monitoring should occur at least four times a year for children and at least yearly for adults. Measurement of white blood cell cystine levels is critical for dose adjustments. Children should be evaluated for growth and nutrition abnormalities and renal function should be closely monitored. Annual eye examinations are required starting in childhood and close monitoring of extrarenal involvement should occur at each visit. Adults should undergo regular neurologic, muscular, and pulmonary evaluations in addition to routine cystine level, graft health, and ophthalmologic monitoring. During the transition from childhood to adulthood, there must not be a lapse in monitoring. Figure modified from Wilmer et al. (10).

Infantile cystinosis is typically diagnosed around 1–2 years of age. Once diagnosed, patients require strict follow-up (Figure 2) (10). WBC cystine levels must be monitored in order to adjust Cysteamine dosage. Growth and nutrition must be closely monitored as well as renal function and electrolytes. Eye examinations should occur annually and neurocognitive monitoring should be in place for patients as well (8, 10). As the patient ages, extrarenal manifestations of the disease must be monitored and cared for as they appear. Close watch over thyroid, pancreatic, pulmonary, gastrointestinal, and neurologic function must occur and treated accordingly (10). This leads to the requirement of a multidisciplinary team in order to fully care for the patient. Cognitively, patients will also require non-medical support as they age in areas of educational and vocational planning (10, 14). As such, a multidisciplinary team must include medical and non-medical personnel with close involvement of family and community members.

## Importance of Transition

Given the multisystem nature, complexity of medical treatment, and multidisciplinary approach required for monitoring and treatment of cystinosis, transition into adulthood is extremely important. The transition period has been identified by patients, families, and physicians as a critical period in the course of cystinosis (14). During this time, families and physicians begin to take a step back and allow the AYA more autonomy and self-reliance as they begin to enter adulthood (2). While AYA’s take on these new medical responsibilities, they are also subjected to the psychosocial, educational, and vocational responsibilities associated with adulthood (15). This influx of responsibilities coupled with psychosocial growth can lead to non-adherence and subsequent progression of disease and loss of transplant as well as poor educational/vocational outcomes, social implications, and mental health problems (10, 15).

As such, patients, families, and physicians have called for a structured transition protocol in order to assist with this critical time (2, 3). A structured transition protocol beginning in early adolescence and concluding in adulthood would help patients ease into their responsibilities. By providing structure, we create a safety net for the patients as they explore their new-found freedoms and responsibilities. This will promote a healthy learning environment and help patients gain confidence and knowledge while helping families “let go” of their loved ones (14). The involvement of a multidisciplinary medical team will help to coordinate new physicians and provide a sense of continuity for the patients. This team will also help address not only the medical issues that arise during transition but the psychological as well.

Recommendations for the comprehensive care of cystinosis were made based on the consensus meetings involving multidisciplinary health-care professionals and literature review (16). These recommendations are based on multidisciplinary team involvement, medication adherence, encouraging patient self-care, and monitoring of polymorphonuclear cystine levels (16). Recently, same group of researchers proposed coordinated transition model for cystinosis with nephrologist as a key player due to high prevalence of chronic kidney disease among cystinosis patients and need for renal transplantation (17). This transition model involves patient and family interests, identifying suitable adult facility and adult nephrologist at appropriate geographical location, establishing communication between pediatric and adult health-care facility with a specific transition plan, follow-up, and monitoring of transition after actual transfer (17). A detailed medical report from pediatric nephrologist consisting of relevant history, diagnosis, treatment, and final visit summary with recommendations should also be sent to receiving adult facility (17). This model also proposes specific transition recommendations to adult departments such as nephrology, neurology, ophthalmology, and endocrinology apart from recommendations for medication adherence and patient self-care (17).

Children with chronic diseases require special health care needs, which are complex and expensive (18). Establishing structured transition program especially in freestanding children hospitals will be faced with operational and financial challenges. Studies investigating the financial implications of running transition programs are lacking. Setting up transition clinic involving multidisciplinary team under one roof increases cost of health care and the operating expenses in teaching hospitals are considerably higher compared to non-teaching community hospitals (18). However, structured transition program aims to reduce the risk of disease complications and graft failure rates thereby reducing long-term medical care costs. A study involving renal transplant recipient children tested the effect of structured transition program with multidisciplinary approach and found that transition program reduced the rate of decline in renal functions and acute rejection episodes compared to control group (19). We outline below our structured transition protocol for cystinosis.

## Transition Protocol

We have developed a transition protocol, RISE to transition, to assist in the transition of AYA’s with Cystinosis. RISE focuses on four key competency areas:

1. *Recognition* of one’s disease process, treatment, health-care system, as well as personal goals, i.e., what is cystinosis and how is it treated? What are barriers to your health access? What are your long-term goals in regards to health, career, and social aspects?
2. *Insight* into short- and long-term impact of their disease, treatment, and non-adherence as well as their social, educational/vocational goals, and other adolescent responsibilities, i.e., why must you be compliant with your treatment? Why is emphasis placed on long-term goals in all aspects of life? Why is there an emphasis placed on this transition period?
3. *Self-reliance and self-management* of medications and appointments as well as be able to identify urgent/emergent changes in their health, i.e., how will you take responsibility for all aspects of your health? How does treatment impact your body and mind? How does your disease effect your social perceptions by yourself and your peers? How will your disease impact your health, career, and social goals?
4. *Establishment of* healthy lifestyle choices, life-long adherence to medications and follow-up, educational/vocational goals, and social responsibilities, i.e., what must you do to live a healthy lifestyle? What can you do to increase adherence to treatments, plans, and appointments? What are your safety nets, sources of support, and facilitators of your medical, social, and educational goals?

These four areas have been identified as key to a successful transition in chronic diseases. Recognition and insight aim to improve patient knowledge of all areas of their disease, including non-medical aspects (9, 20). Self-reliance and establishment aim to improve patient autonomy and build toward a lifetime of success in regards to disease management as well as all dreams and aspirations the patient may have (9, 14, 15). Proficiency in all four areas will allow the patient to RISE through transition and gain the skills to be successful in the adult medical world and beyond.

Identifying the areas is not enough, we must be able to assess and monitor competency as well as assist the patient as he/she moves through this period (20). In order to do this, our protocol calls for the use of standardized transition readiness surveys, medical passports, adherence monitoring, and other readily available transition tools (21). We also call for a dedicated transition clinic. This will be a multidisease clinic, where the focus is the transition of multiple chronic diseases rather than just cystinosis. One day each month will be dedicated for cystinosis and all medical personnel from adult and pediatric sides will be in attendance. This will allow for close collaboration between teams as well as provide patients the convenience of a 1-day visit (14).

### Personnel Involved in Transition

**Figure 3:**
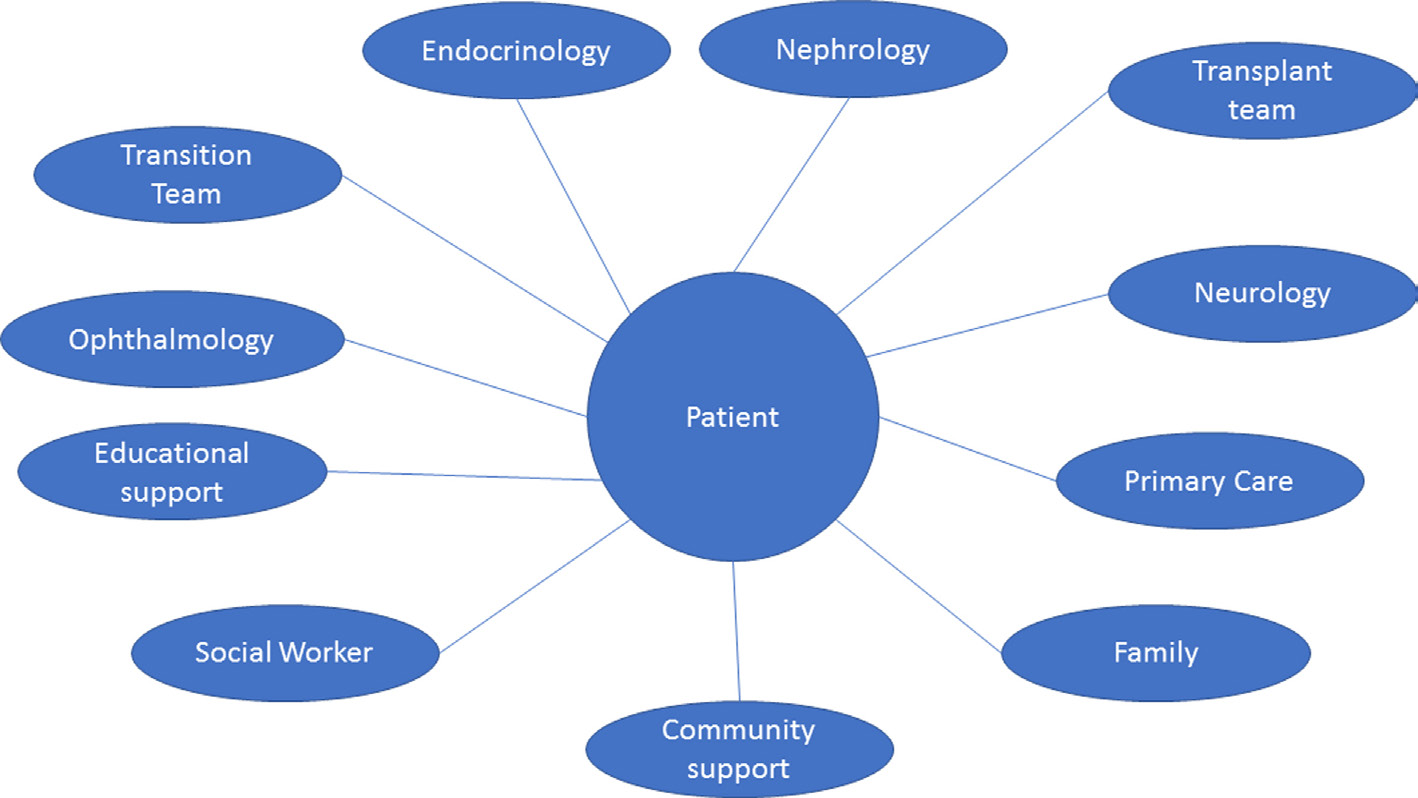
Multispecialty and multidisciplinary approach. The multisystem nature of cystinosis presents a wide range of clinical and non-clinical challenges. As such, management requires the coordination of multiple specialties, health-care providers, community support, and many other personnel. The ideal multidisciplinary team for transition should include: adult and pediatric nephrology, endocrinology, transplant team, ophthalmology, pediatrician and/or primary care physician, and as needed neurology, cardiology, and other specialties. Support should be in place from a pharmaceutical, community, educational, and vocational sectors of the patient’s life.

The complex nature of cystinosis requires a multidisciplinary team (Figure 3). It will include:

(1) Pediatric/adult nephrology
(2) Transition team/clinic
(3) Pediatric/adult transplant teams
(4) Primary care physician (PCP)
(5) Transition coordinator
(6) Community services (will cover educational/vocational aspects)
(7) Pediatric/adult ancillary services as needed (i.e., endocrine, pulmonology, GI, etc.)
(8) Adolescent medicine and obstetrics and gynecology
(9) Social work/pharmacy/psychologist/counselors/dietician/occupational therapy specialist.

These will be the key team members involved during transition (14, 22). Pediatric nephrology will be the primary team prior to and during transition. During transition, primary responsibilities will shift to the adult nephrologist as well as the patient’s chosen PCP. This gradual transition will allow the patient time to acclimate to his/her new environment as well as allow the PCP to be “brought up to speed” with the patient’s complex medical care (23).

The transition team/clinic will be the primary location for medical appointments during transition. This will provide a single location for patients to meet with their entire medical team.

We plan to have a dedicated “Cystinosis Day” for patients. This day would include all services in one location on 1 day. This strategy has been identified by patients as something would be beneficial to their care (14). From a medical team standpoint, this would allow for easier collaboration between teams across different medical specialties.

### Stages of Transition

**Figure 4:**
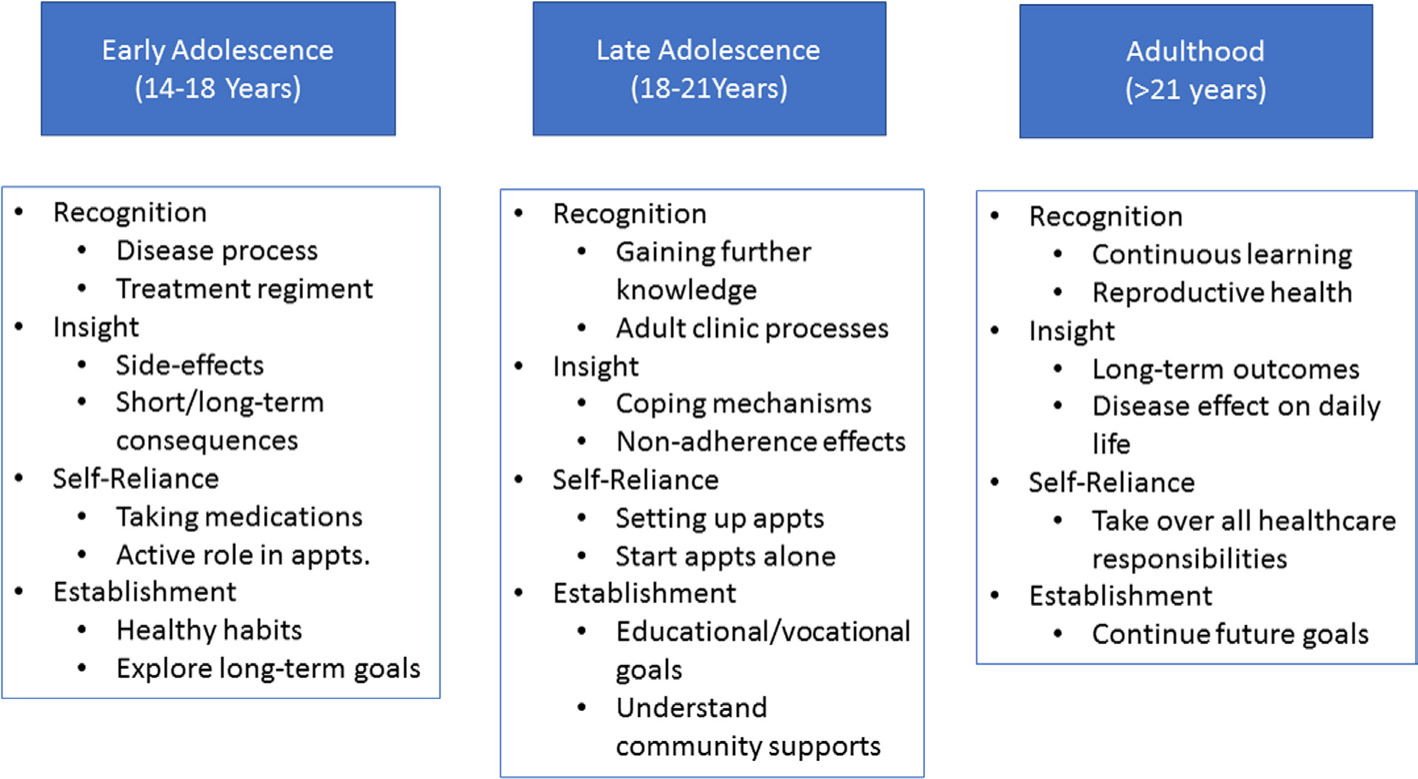
Stages of transition. Each stage (age group) of transition is associated with its own set of Recognition, Insight, Self-reliance, and Establishment of healthy habits (RISE) expectations. Pretransition (early adolescence) is focused on basic disease and medication knowledge acquisition, begin independent skill acquisition, and establishing life-long goals and habits. During the active transition stage (late adolescence) further emphasis will be placed on independent skills as well as defining and working toward educational/vocational goals. The post-transition phase (adulthood) will focus on continuous acquisition of knowledge, skills, and independence.

**Figure 5:**
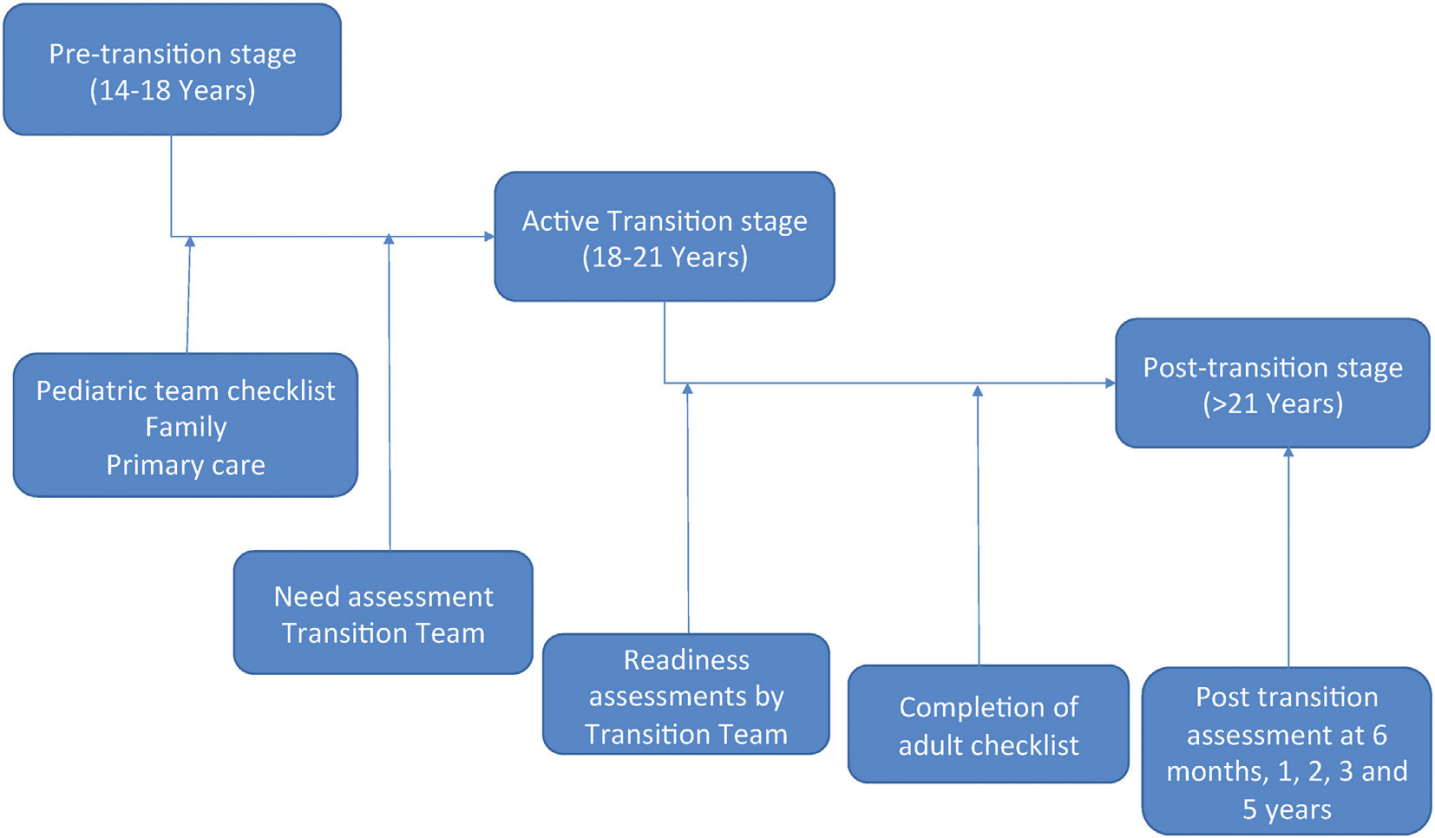
Transition flow. The pretransition stage begins between 14 and 18 years of age. At this time, the pediatric team will be provided a checklist and will begin preparing the patient for active transition. This will include: completing the checklist, assessing Recognition, Insight, Self-reliance, and Establishment of healthy habits (RISE) competencies, ensuring family and primary care physician involvement, and other tasks. Once the team determines the patient is ready, a need assessment will be completed by the transition team and the patient will enter the active transition stage. During this stage, patients will work closely with the transition, adult, and pediatric teams in order to address any deficiencies noted during the need assessment. This will be a gradual process that occurs between 18 and 21 years of age. Throughout the active transition stage, readiness assessments will be conducted by the transition team. Once the patient has completed all checklist items and is deemed competent in all RISE areas, the patient will enter the posttransition stage. This will occur by 21 years of age and will have continuous monitoring by the transition team in order to determine outcome.

There are three stages to transition (Figure 4). At each stage, patients will be expected to reach certain RISE milestones and will be assessed regularly (Figure 5).

#### Pretransition Stage

This will occur between 14 and 18 years of age. During this time, the patient will be primarily cared for by the pediatric nephrology team and family with support from all other specialties.

#### Active Transition Stage

Transition will occur between the age range of 18–21 years. During this time, there will be a gradual shift in leadership from the pediatric nephrology team and family to the patient, PCP, and adult nephrology team. All medical services will also transfer to adult care services. This transition will occur gradually with the use of a multidisciplinary team.

#### Posttransition Stage

This stage will occur after the age of 21. The patient, PCP, and adult nephrology team will take over primary medical responsibilities with support from all other medical services. The transition team will remain on as support during the initial posttransition years.

### Stage 1: Pre-Transition (14–18 Years)

Transition will begin in early adolescence and will be headed by the pediatric nephrologist. As nephrology tends to be the first specialty involved in patient care, they will be the point of contact for transition as well. Starting in early adolescence, the pediatric team will begin laying the foundation for transition. The pediatric team will begin educating the patient in the four competency areas.

(1) *Recognition*: Pediatric team will educate patient on basic knowledge of his/her disease and treatment.
(2) *Insight*: Pediatric team will educate patient on short/long-term outcomes related to disease and treatment, side effects along with coping mechanisms.
(3) *Self-reliance*: Pediatric team will lay the groundwork for self-reliance by promoting a greater patient led role during visits. Families will be encouraged to start giving patients more autonomy.
(4) *Establishment of healthy habits*: Pediatric team with the help of family will encourage a healthy lifestyle, advocate for future educational/vocational planning, and life-long adherence to treatments.

The pediatric team will begin laying the foundation for these goals around the age of 14. In order to assist the team, a checklist of milestones will be provided to the pediatric team (Table 1). Ideally, the patient will have reached all milestones on this checklist prior to transition. The pediatric team will also ensure the patient has a PCP at the initiation of active transition. The PCP is an important piece to transition. As the patient advances into adulthood, the PCP takes on a more central role in medical management. As such, PCPs need to be included early on and begin building a relationship with the patient, family, and entire medical team (23). To assist with early knowledge acquisition and begin fostering self-reliance, a health-care passport will be provided to the patient by the pediatric team.

**Table 1:** Milestone checklist (pretransition stage).

Prior to initiating transition, the pediatric team will ensure the patient is competent in the following areas:
*Recognition* ◻ Disease pathology and manifestations ◻ Treatment regimen ◦ Medications and what they are for ◦ Frequency and route of administration ◻ Their role in disease management ◻ Short- and long-term educational/vocational goals

*Insight* ◻ Short- and long-term health outcomes related to their disease and treatment ◻ Medication side effects ◦ Minimizing or coping with side effects ◻ Consequences of medication non-adherence ◻ How the disease will affect educational/vocational goals ◻ How the disease will affect social aspects of their lives

*Self-Reliance* ◻ Be able to consistently take medications ◻ Attend, participate, and act independently during appointments ◻ Call in for refills and appointments ◻ Recognize urgent/emergent changes in their health

*Establishment of health habits* ◻ Lifelong healthy choices ◻ Continuous adherence to treatment regimen ◻ Continuous pursuit of educational/vocational goals ◻ Continuous acceptance of responsibilities as he/she enters adulthood

Given the challenges children with cystinosis face, future planning must also be part of the transition process. As such, the pediatric team will encourage the patient and family to begin planning for his/her educational or vocational future (9). At the same time, the team will encourage the patient and family to seek out community support in order to address the social challenges faced by the patient as he/she approaches adulthood (9). By starting early, we hope to have established community support by the time the patient reaches active transition.

In order to assist in the acquisition of knowledge and skills, a milestone checklist will be provided to the pediatric team. Each competency consists of four to five skills the patient should acquire prior to transition. In order to pass onto active transition, the pediatric team must confirm the patients have achieved proficiency in at least 50% of the skills in each proficiency area. It will be up to the pediatric team’s discretion on which skills will be focused on during the pretransition phase. This degree of freedom will allow the pediatric team to focus on areas where they feel the patient is most comfortable or most capable. By taking this approach, we hope to build the patient’s confidence and reinforce his/her abilities prior to reaching the transition period.

Once the pediatric nephrology team deems the patient ready for transition, they will reach out to the adult nephrology team, transition team/clinic, and PCP and initiate the transition process. They will also prepare a written transition plan to be given to the patient, family, and adult team.

### Stage 2: Active Transition (18–21 Years)

#### Need Assessment

Once transition is initiated, the patient will enter the active transition phase. The first step in this stage will be to assess the patient’s baseline knowledge and readiness. This baseline assessment will help determine level of need as well as personalize the transition process for the patient.

#### Active Transition

The first visit will always be a combined visit involving pediatric, adult, and transition teams. This visit will occur at the transition clinic. During this visit, the pediatric nephrology team will present the patient, family, and adult team with a written transition plan. This plan will provide the structure for transition and will be adjusted based on the baseline assessment and how the patient progresses through transition. At this time, the adult team will also lay out their expectations from the patient. This will include things such as clinic procedures, medication, and appointment requests, and any other rules/requirements by the adult team.

Because of the multisystem nature of cystinosis, this will occur with all specialties. Given the nature of cystinosis, the pediatric nephrologist typically is the first to diagnose the disease and has been with the child since early childhood. Therefore, the initial visit will always include the pediatric and adult nephrology teams. The transplant teams will always be included in the initial visit as well due to the tenuous nature of renal function in cystinosis. Depending on the number of specialties currently involved, the initial visit may be divided into multiple days in order to allow the patient time to process each team’s expectations.

The transition team will consist of a multidisciplinary team including med-peds personnel, transition coordinators, social workers, pharmacy, and other needed personnel. The team will also include community and educational/vocational representatives in order to help the patient transition from a social standpoint. The transition team will also be responsible for the continuous assessment of readiness (14, 22). Various validated readiness surveys will be conducted every 6 months to assess the patients’ progress. Based on the results, transition will be adjusted for the patient’s weaknesses. The team will also monitor the use of the health-care passport and make adjustments as necessary throughout the process.

Emphasis will also be placed on the psychological health of the patient. This has been an area identified by patients, family, and health-care professionals as an area of importance. We believe our structured protocol, dedicated personnel, and incorporation of medical and community support will help patients feel more included in the medical decision making and help normalize his/her disease. The incorporation of community support will help give the patient a better sense of “normal” and provide help outside of the medical community. Psychological support will be present to help the patient learn to cope, adapt, and self-manage their minds, bodies, and souls throughout the process. Families will be included in order to provide further safety nets and community support will help the patient find a sense of normalcy (14). Educational and social support will also be provided in order to help the patient progress to their educational/vocational goals.

The adult nephrology team will be provided a milestone checklist covering the four competency areas (Table 2). The adult team will continue to build on the foundation created by the pediatric team and begin incorporating more responsibilities and knowledge of adult medicine. The transition team will assist in the assessing the patient’s readiness and competency. They will also assist in coordinating education between the different specialties in order to address patient weaknesses. This is especially important in the case of cystinosis given its need for a multispecialty approach.

**Table 2:** Milestone checklist (active transition stage).

Prior to completing transition, the transition team and adult nephrology team will ensure the patient is competent in the following areas:
*Recognition* ◻ Disease pathology and manifestations ◻ Treatment regimen ◦ Medications and what they are for ◦ Frequency and route of administration ◻ Their role in disease management ◻ Short- and long-term educational/vocational goals ◦ Recognition of tools, advocates, and programs at their disposal ◻ Adult clinic responsibilities and expectations ◻ Insurance and financial responsibilities ◻ Awareness of social responsibilities

*Insight* ◻ Short- and long-term health outcomes related to their disease ◻ Medication side effects and ways to minimize them ◻ Consequences of non-adherence ◻ How the disease will affect educational/vocational goals ◻ How the disease will affect social aspects of their lives ◦ Be able to seek appropriate resources ◻ Navigation of insurance and financial responsibilities

*Self-reliance* ◻ Be able to consistently take medications ◻ Attend, participate, and act independently during appointments ◻ Call in for refills and appointments ◻ Recognize urgent/emergent changes in their health ◻ Financial responsibility ◻ Social responsibility

*Establishment of health habits* ◻ Lifelong healthy choices ◻ Continuous adherence to treatment regimen ◻ Continuous pursuit of educational/vocational goals ◻ Continuous acceptance of responsibilities as he/she enters adulthood

By the end of the active transition period, the adult and transition teams will ensure the patient has reached all milestones and are competent in all four areas. The team will ensure the patient:

(1) *Recognition*: Understands the responsibilities of the adult clinic, knowledge of disease process, knowledge of treatment regimen.
(2) *Insight*: Understands short-/long-term outcomes related to disease, treatment, and non-adherence. Understand how their disease/treatment effects social, educational, and vocational aspects of their lives.
(3) *Self-reliance*: The patient will be able to schedule his/her own appointments, take his/her own medications as scheduled, request refills, recognize emergent/urgent changes in health status, and manage his/her educational/vocational goals.
(4) *Establish healthy habits*: The patient will establish healthy lifestyle choices, life-long adherence to medications and appointments, educational/vocational goals, social responsibilities, and willingness to seek assistance as needed.

The adult nephrology team as well as the transition team will work closely to assess the patient’s readiness for transition. Once all milestone checklist skills have been acquired, the patient will be considered ready for transition. At this point, the team will finalize transition and confirm the following:

(1) PCP is aware of the patient’s disease process, treatment, and specialists involved.
(2) All specialties are in agreement that the patient is ready to complete transition.
(3) All specialties have set up a follow-up appointment.
(4) Appropriate community support has been established.

### Stage 3: Post-Transition (>21 Years)

After the patient has been fully transitioned, the PCP and adult teams will take over full medical responsibilities. The patient will fully take over his/her own medical care at this time and be responsible for his/her actions and consequences. Each pediatric team will remain with the patient until the first official adult visit. After that point, the team will be available for consultation on an as needed basis for the adult team. As a safety net, the family will remain available to assist the patient as he/she progresses into adulthood. The transition team will also remain with the team in order to provide support as well as perform quality assessment in order to improve the transition process. At 6 months, posttransition, a combined teleconference will be conducted with all personnel involved to assess the quality of transition. After three consecutive visits with adult providers, quality outcome will be initiated with assessments at 1, 2, 3, and 5 years posttransition. We also hope that patients and families that experience a positive transition through the process will bring their wisdom and insight back into the transition clinic and act as mentors and advocates for new patients.

## Conclusion

Adolescents and young adults with chronic diseases such as cystinosis face a serious challenge as they reach adulthood. The need for a structured transition has been called for by the Cystinosis Research Network. With this structured protocol, we hope to improve the quality of life of cystinosis patients and ease the growing pains associated with emerging adulthood. Our goal is to not only improve transition from a medical perspective but also from a social, psychological, and personal perspective. Our protocol aims to achieve this goal with the use of a multidisciplinary medical team along with validated transition assessment tools. This structured protocol will also allow for further transition research and improvement of the process for future patients.

## Author Contributions

RR, JW, and VK were responsible for concept and design of this protocol. All the authors contributed to drafting and revising the manuscript and approved the final version to be published.

## Conflict of Interest Statement

The authors declare that the research was conducted in the absence of any commercial or financial relationships that could be construed as a potential conflict of interest.
